# A systematic review and meta-analysis of the prevalence of thrombosis and bleeding at diagnosis of Philadelphia-negative myeloproliferative neoplasms

**DOI:** 10.1186/s12885-019-5387-9

**Published:** 2019-02-28

**Authors:** Tarinee Rungjirajittranon, Weerapat Owattanapanich, Patompong Ungprasert, Noppadol Siritanaratkul, Theera Ruchutrakool

**Affiliations:** 1 Division of Medicine, Phranangklao Hospital, Nonthaburi, 11000 Thailand; 2 Division of Hematology, Department of Medicine, Faculty of Medicine Siriraj Hospital, Mahidol University, 2 Wanglang Road, Bangkok, 10700 Thailand; 3 Clinical Epidemiology Unit, Department of Research and Development, Faculty of Medicine Siriraj Hospital, Mahidol University, Bangkok, 10700 Thailand

**Keywords:** Myeloproliferative neoplasms, Polycythemia vera, Essential thrombocythemia, Primary myelofibrosis, Prevalence, Thrombosis, Bleeding, Hemorrhage

## Abstract

**Background:**

Philadelphia (Ph) chromosome-negative myeloproliferative neoplasms (MPNs) are a heterogeneous group of hematopoietic stem cell clonal diseases. Most patients with MPN are asymptomatic at diagnosis although some of them suffer from constitutional symptoms. Thrombosis and bleeding can also be one of the initial manifestations although the reported prevalence varied considerably across the studies. This systematic review and meta-analysis was conducted with the aims to better understand the prevalence and characteristics of thrombosis and bleeding among patients with newly-diagnosed MPN.

**Methods:**

Using a search strategy that included the terms for myeloproliferative neoplasms, thrombosis, and bleeding, two investigators independently searched for published articles indexed in the MEDLINE and EMBASE databases from inception to August 2018. The pooled prevalence was calculated using the DerSimonian–Laird random-effects model with a double arcsine transformation.

**Results:**

A total of 29 cohort studies (8 prospective and 21 retrospective) with 13,436 patients with MPN were included into this meta-analysis. At diagnosis, the pooled prevalence of overall thrombosis among patients with MPN was 20.0% (95% CI, 16.6–23.8%; I^2^ 96%), with the pooled prevalence of arterial thrombosis of 16.2% (95% CI, 13.0–20.0%; I^2^ 95%) and the pooled prevalence of venous thrombosis of 6.2% (95% CI, 4.9–7.8%; I^2^ 89%). Common thrombotic events included cerebrovascular disease/transient ischemic attack, coronary heart disease, and deep venous thrombosis. The pooled prevalence of hemorrhagic complications among patients who were newly diagnosed with MPN patients was 6.2% (95% CI, 5.0–7.8%; I^2^ 85%). Common sites of bleeding included gastrointestinal, mucosal, and cutaneous bleeding.

**Conclusions:**

Thrombosis and bleeding are common initial manifestations of MPN. Investigations for MPN should be considered for patients who present with unexplained thrombosis or abnormal bleeding.

**Electronic supplementary material:**

The online version of this article (10.1186/s12885-019-5387-9) contains supplementary material, which is available to authorized users.

## Background

Philadelphia (Ph) chromosome-negative myeloproliferative neoplasms (MPNs) are a heterogenous group of hematopoietic stem cell clonal diseases, of which the main subtypes are polycythemia vera (PV), essential thrombocythemia (ET), and primary myelofibrosis (PMF) [1, 2]. More than a half of the patients are asymptomatic at diagnosis although some of them suffer from weight loss, fatigue, fever, pruritus, and early satiety. Thrombosis and bleeding can also be ones of the initial manifestations that eventually lead to the diagnosis of MPN [3, 4].

Thrombosis in patients with MPN could manifest as mild microcirculatory disturbance or as major arterial and venous thrombotic events such as ischemic stroke, myocardial infarction, peripheral arterial disease, and deep vein thrombosis [5]. Similarly, bleeding in these patients can be a minor one or could manifest as major internal organ hemorrhage [6]. The reported prevalence of thrombosis and bleeding among patients who were newly diagnosed with MPN varied considerably across the studies [7–35]. As one of the aims of the treatments of MPN is to decrease the risk of thrombosis and some prescribed medications, such as aspirin, can increase the risk of bleeding, knowing the baseline prevalence of thrombosis and bleeding would be of clinical importance for clinicians who need to balance the risk between these two opposite complications. The current systematic review and meta-analysis was conducted with the aims to better understand the prevalence and characteristics of thrombosis and bleeding among patients with newly-diagnosed MPN by comprehensively identifying all available studies and summarizing their results together.

## Methods

### Data sources and searches

Using a search strategy that included the terms for myeloproliferative neoplasms, thrombosis, and bleeding, two investigators (T.R.1 and W.O.) independently searched for published articles indexed in the MEDLINE and EMBASE databases from inception to August 2018. The search strategy is available as Additional file 1. In addition, the references of the included studies were also manually reviewed to identify additional eligible studies. This study was performed according to the Preferred Reporting Items for Systematic Reviews and Meta-Analyses statement, which is available as Additional file 2 [36].

### Selection criteria and data extraction

To be eligible for inclusion into the meta-analysis, first, the study needed to consist of at least one cohort of patients who were newly diagnosed with Philadelphia negative MPNs (PV, ET, or PMF). Then, the study needed to report the overall prevalence of thrombosis and/or bleeding at diagnosis of that cohort. The secondary outcomes of interest, including prevalence of thrombosis for each MPN subtype, prevalence of thrombosis at each location, prevalence of bleeding for each MPN subtype, and prevalence of bleeding at each location, were also collected for pooled analysis but were not part of the inclusion criteria. Both investigators evaluated all studies independently. If different decisions regarding the eligibility of a study were made, the study in question was jointly reviewed by the two investigators and the final determination was reached by consensus.

### Statistical analysis

Comprehensive Meta-Analysis program, version 2.2 (Biostat, Englewood, NJ, USA) was used to analyze all data. The same two authors (T.R.1 and W.O.) extracted and tabulated all data from each eligible study using a standardized data extraction form. The pooled rates and 95% confidence intervals (CI) of the overall prevalence of thrombosis at diagnosis, overall prevalence of bleeding at diagnosis, prevalence of thrombosis at each location, prevalence of bleeding for each MPN subtype, and prevalence of bleeding at each location, were calculated using the DerSimonian–Laird random-effects model with a double arcsine transformation [37]. The random-effects model was utilized as opposed to a fixed-effects model as the between-study heterogeneity was suspected to be high due to the difference in background populations between studies. The heterogeneity was calculated using Cochran’s Q test and the I^2^ statistic. The I^2^ statistic quantifies the proportion of total variation across studies; the I^2^ values were classified as follows: an I^2^ of 0–25% indicated insignificant heterogeneity; 26–50%, low heterogeneity; 51–75%, moderate heterogeneity; and greater than 75%, high heterogeneity [38].

## Results

**Fig. 1:**
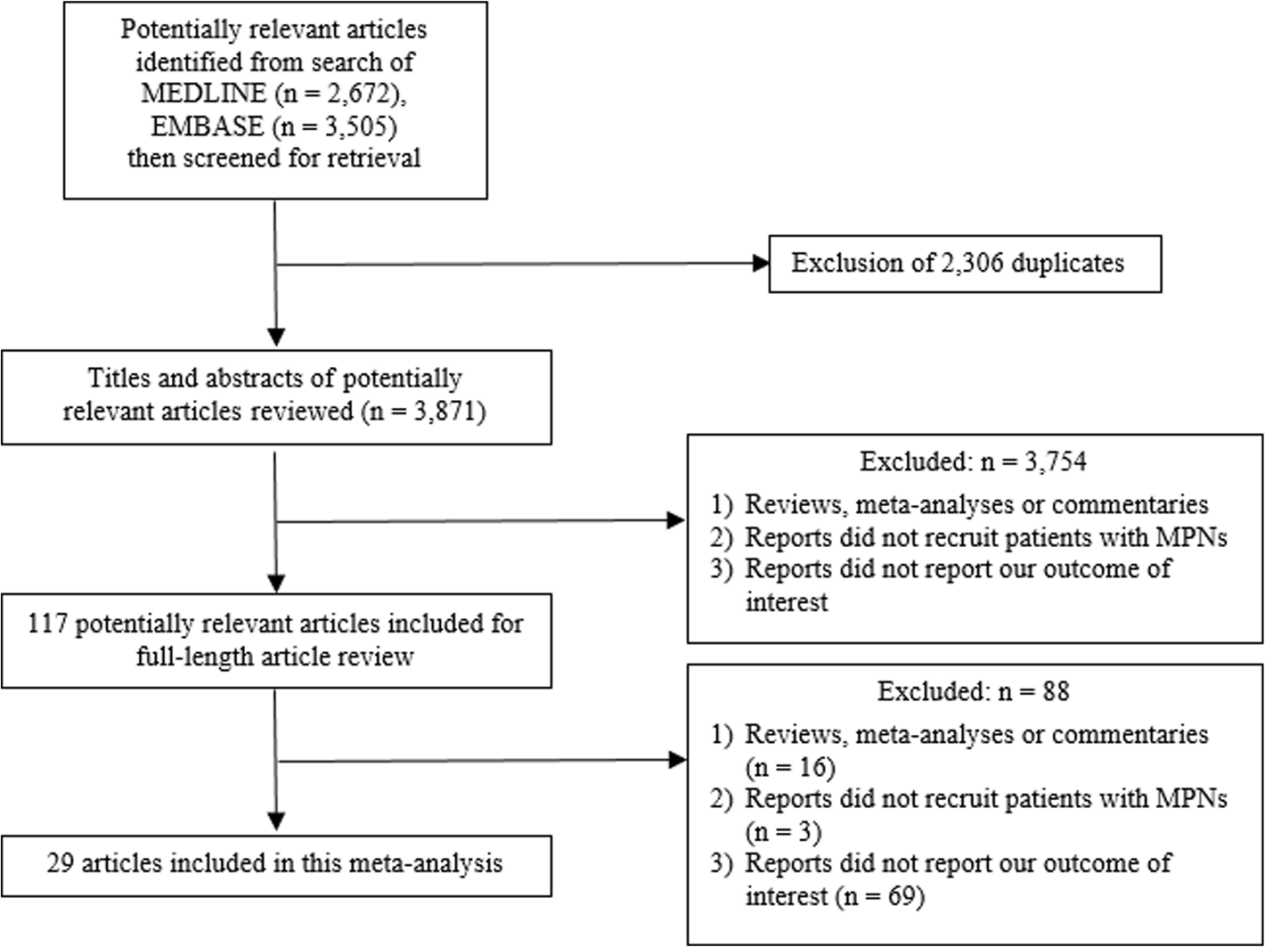
Flowchart of literature review process

A total of 6177 articles (2672 from MEDLINE and 3505 from EMBASE) were identified using the aforementioned search strategy. A total of 2306 articles were duplication between the databases, which were removed using EndNote X8 software, leaving 3871 articles for the first round of review (review of title and abstract). After the first round of review, 3754 articles were excluded because they clearly did not meet the pre-specified inclusion criteria based on type of article, study design, and study participants. A full-text review of the remaining 117 articles was undertaken and 88 articles were found to be ineligible for the meta-analysis (16 articles were reviews, meta-analysis or commentaries; 3 articles did not recruit patients with MPNs; and 69 articles did not report our outcome of interest). Finally, 29 cohort studies (8 prospective studies and 21 retrospective studies) were included in the meta-analysis [7–35]. The literature review and identification process are summarized as Fig. 1.

### Baseline patient characteristics

A total of 13,436 patients who were newly diagnosed with MPN from 29 studies were included in this meta-analysis. ET was the most common subtype of MPN among patients analyzed in this meta-analysis (49.2%), followed by PV (34.7%), and PMF (14.4%). There was a slight overall female predominance (52.4%) while the median age of participants ranged from 42 to 76 years across the studies. The prevalence of thrombosis was reported by 26 studies while the prevalence of bleeding was reported by 21 studies. Table 1 describes the characteristics and participants of the included studies. The data on managements and clinical outcomes of participants in these studies are described in Additional file 3: Table S1.

**Table 1:** Baseline patient characteristics of the included studies

References	No.	Sex (M/F)	Median age (years, range)	No. of PV	No. of ET	No. of PMF	Thrombotic outcome	Bleeding outcome	Study period	Type
Fenaux 1990 [7]	147	60/87	60 (18–83)	–	147	–	Yes (A + V)	Yes	1970–1987	R
Colombi 1991 [8]	103	44/59	59 (9–88)	–	103	–	Yes (A + V)	Yes	1975–1990	R
Besses 1999 [9]	148	53/95	60.5 (11–85)	–	148	–	Yes (A + V)	Yes	1979–1994	R
Manoharan 1999 [10]	61	PV: 10/ 10 ET: 10/ 20 PMF: 5/ 6	PV: 76 (54–84) ET: 71 (25–88) PMF: 74 (31–84)	20	30	11	Yes (A + V)	No	1993–1997	P
Jensen 2000 [11]	96	27/69	67 (18–87)	–	96	–	Yes (A + V)	Yes	1977–1998	R
Passamonti 2000 [12]	163	98/65	57 (30–82)	163	–	–	Yes (A + V)	Yes	1975–1997	R
Chim 2005 [13]	231	112/119	65 (18–90)	–	231	–	Yes (A + V)	Yes	NR	R
Marchioli 2005 [14]	1638	942/ 696	60.4	1638	–	–	Yes (A + V)	Yes	NR	P
Cervantes 2006 [15]	155	97/ 58	65 (17–89)	–	–	155	Yes (A + V)	No	1972–2005	R
Wolanskyj 2006 [16]	322	104/ 218	54 (12–88)	–	322	–	Yes (A + V)	Yes	1956–1992	R
Carobbio 2007 [17]	439	175/ 264	54 (10–93)	–	439	–	Yes (A + V)	No	1981–2006	R
Vannucchi 2007 [18]	962	PV: 176/ 147 ET: 202/ 437	NR	323	639	–	Yes (A + V)	No	NR	R
Bang 2009 [19]	283	143/ 140	61	120	NR	NR	Yes (A + V)	Yes	2006–2007	P
Barbui 2010 [20]	707	465/ 242	62 (11–90)	–	–	707	Yes (A + V)	No	1973–2008	R
Elliott 2010 [21]	205	131/ 74	62 (28–87)	–	–	205	Yes (A + V)	No	1982–2008	R
Palandri 2011 [22]	532	205/ 327	64 (16–95)	–	532	–	Yes (A + V)	No	1978–2008	R
Buxhofer-Ausch 2012 [23]	264	109/ 155	57.4	–	–	264	Yes (A + V)	No	NR	P
Finazzi 2012 [24]	1104	ET: 370/ 521 PMF: 74/ 106	ET: 55.7 (12.9–91) PMF: 57.4 (20.9–87.7)	–	891	180	No	Yes	NR	P
Angona 2015 [25]	214	57/ 157	64 (9–93)	–	214	–	Yes (A + V)	No	1985–2012	R
Enblom 2015 [26]	612	PV: 131/ 118 ET: 117/ 155 PMF: 47/ 44	PV: 69 ET: 67 PMF: 71	249	272	91	Yes (A + V)	Yes	1995–2013	R
Lim 2015 [27]	102	54/ 48	64 (24–87)	33	69	–	Yes (A)	Yes	2004–2012	R
Duangnapasatit 2015 [28]	157	PV: 46/ 22 ET: 32/ 51 PMF: 4/ 2	PV: 59.6 (18–88) ET: 61.1 (21–89) PMF: 68.3 (52–78)	68	83	6	Yes (A + V)	Yes	2003–2013	R
Kaifie 2016 [29]	454	232/ 222	60	142	140	113	Yes (A + V)	Yes	NR	P
Cerquozzi 2017 [30]	587	284/ 303	60 (17–94)	587	–	–	Yes (A + V)	No	NR	P
Abdulkarim 2017 [31]	2389	PV: 548/557 ET: 534/750	PV: 69 (17–98) ET: 68 (13–94)	1105	1284	–	Yes (A + V)	Yes	2008–2015	R
Soyer 2017 [32]	708	PV: 132/ 81 ET: 151/ 239 PMF: 56/ 49	PV: 47.5 (17–86) ET: 41.5 (17–89) PMF: 69.5 (19–87)	213	390	105	Yes (A + V)	Yes	1987–2014	R
Bertozzi 2017 [33]	253	NR	NR	124	121	8	Yes (A)	Yes	1978–2016	R
Zhou 2018 [34]	150	67/ 83	61 (41–71)	–	150	–	Yes (A + V)	No	2013–2016	P
Hintermair 2018 [35]	250	152/ 98	NR	NR	NR	NR	Yes (A + V)	No	2008–2015	R

*Abbreviations*: *A* Artery, *ET* Essential thrombocythemia, *F* Female, *M* Male, *NR* Not reported, *P* Prospectively, *PMF* Primary myelofibrosis, *PV* Polycythemia vera, *R* Retrospectively, *V* Vein

### Prevalence of thrombosis at diagnosis of MPN

**Fig. 2:**
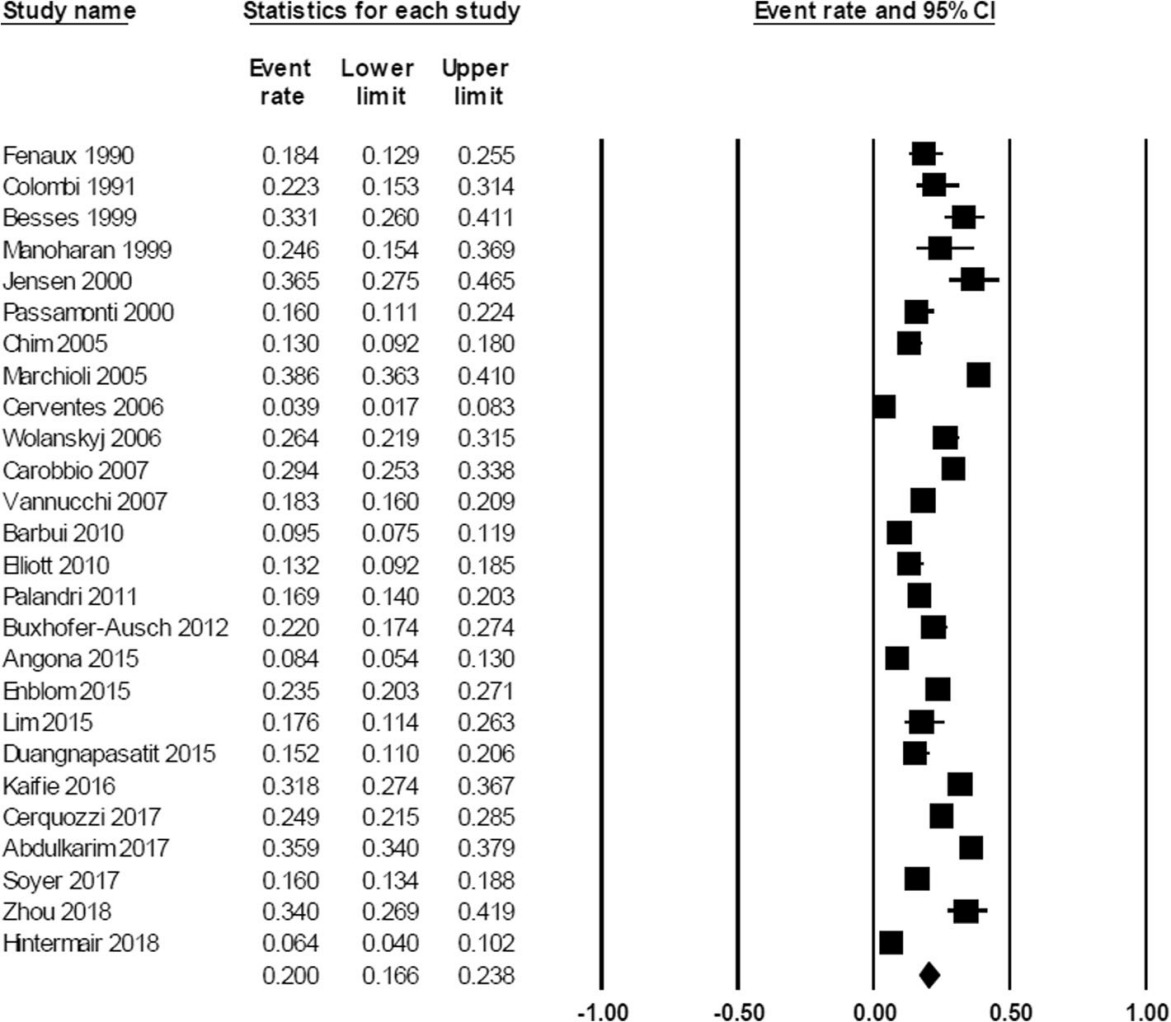
Forest plot of pooled prevalence and 95% confidence interval of overall thrombosis in patients with MPN

**Fig. 3:**
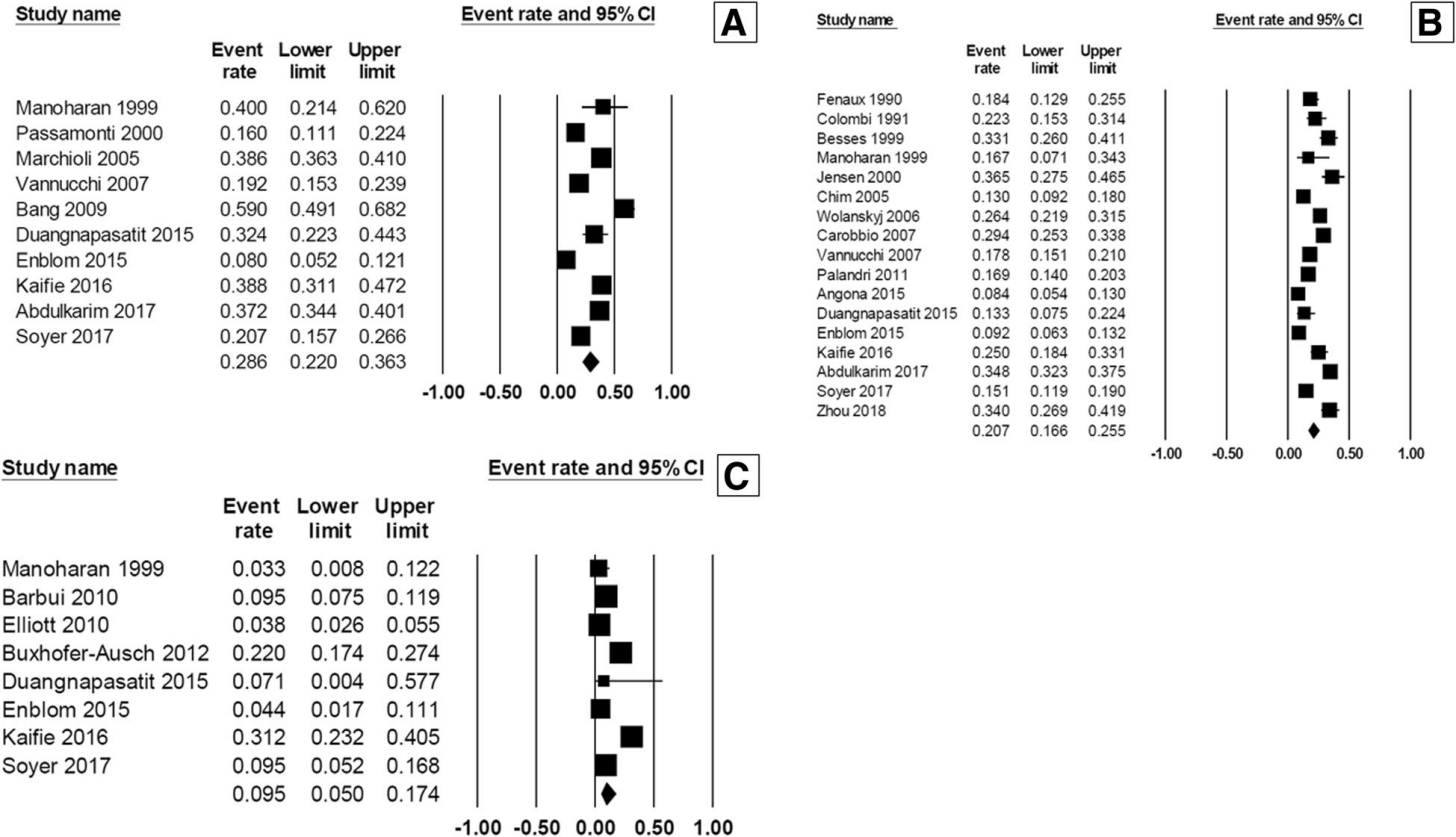
Forest plot of pooled prevalence and 95% confidence interval of overall thrombosis of each MPN subtype: **a** polycythemia vera; (**b**) essential thrombocythemia; (**c**) primary myelofibrosis

**Fig. 4:**
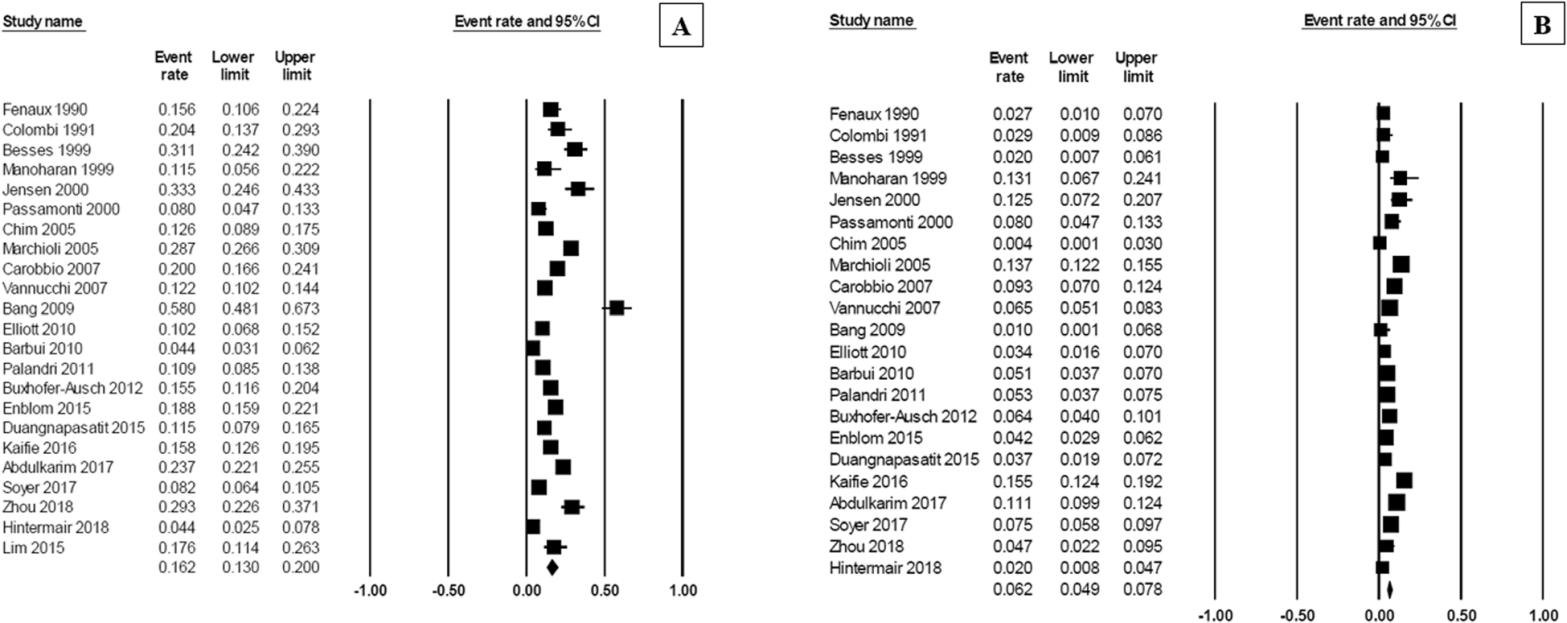
Forest plot of pooled prevalence and 95% confidence interval of (**a**) arterial thrombosis and (**b**) venous thrombosis in patients with MPN

At diagnosis, the pooled prevalence of overall thrombosis (either arterial or venous) among patients with MPN was 20.0% (95% CI, 16.6–23.8%; I^2^ 96%; Fig. 2) [7–18, 20–23, 25–32, 34, 35]. The pooled prevalence for each MPN subtype was as followed; PV 28.6% (95% CI, 22.0–36.3%; I^2^ 95%) [10, 12, 14, 18, 19, 26, 28, 29, 31, 32], ET 20.7% (95% CI, 16.6–25.5%; I^2^ 93%) [7–11, 13, 16–18, 22, 25, 26, 28, 29, 31, 32, 34], and PMF 9.5% (95% CI, 5.0–17.4%; I^2^ 94%) [10, 20, 21, 23, 26, 28, 29, 32] (Fig. 3). The pooled prevalence of arterial thrombosis was 16.2% (95% CI, 13.0–20.0%; I^2^ 95%) [7–14, 17–23, 26–29, 31, 32, 34, 35] while the pooled prevalence of venous thrombosis was 6.2% (95% CI, 4.9–7.8%; I^2^ 89%) (Fig. 4) [7–14, 17–23, 26, 28, 29, 31, 32, 34, 35].

### Sites of arterial thrombosis

The pooled prevalence of arterial thrombosis at diagnosis of MPN for each specific site was as followed; cerebrovascular disease 7.4% (95% CI, 5.0–10.8%; I^2^ 90%) [7–9, 11, 13, 14, 19, 21, 25–27, 29], transient ischemic attack of 3.5% (95% CI, 1.9–6.4%; I^2^ 91%) [8, 9, 11, 13, 19, 21, 25–27, 35], coronary heart disease 6.1% (95% CI, 5.1–7.4%; I^2^ 73%) [7–14, 17, 19–22, 25–29, 31, 35], and peripheral arterial disease 3.3% (95% CI, 2.2–4.8%; I^2^ 87%) [7–9, 11, 13, 14, 17, 19–22, 26, 28, 31]. The forest plots of each arterial thrombotic event are provided as Additional file 4.

### Sites of venous thrombosis

The pooled prevalence of venous thrombosis at diagnosis of MPN for each specific site was as followed; deep vein thrombosis 3.4% (95% CI, 2.0–5.6%; I^2^ 85%) [7–9, 14, 18, 19, 28, 29, 35], splanchnic vein thrombosis 1.4% (95% CI, 0.8–2.2%; I^2^ 78%) [12, 13, 18, 20, 21, 26, 28, 29, 31, 35], pulmonary embolism (PE) 0.9% (95% CI, 0.4–2.3%; I^2^ 74%) [9, 14, 18, 28, 35], and cerebral venous sinus thrombosis 0.7% [95% CI, 0.2–2.3%; I^2^ 0%] [21, 28]. The forest plots of each venous thrombotic event are provided as Additional file 4.

### Prevalence of bleeding at diagnosis of MPN

**Fig. 5:**
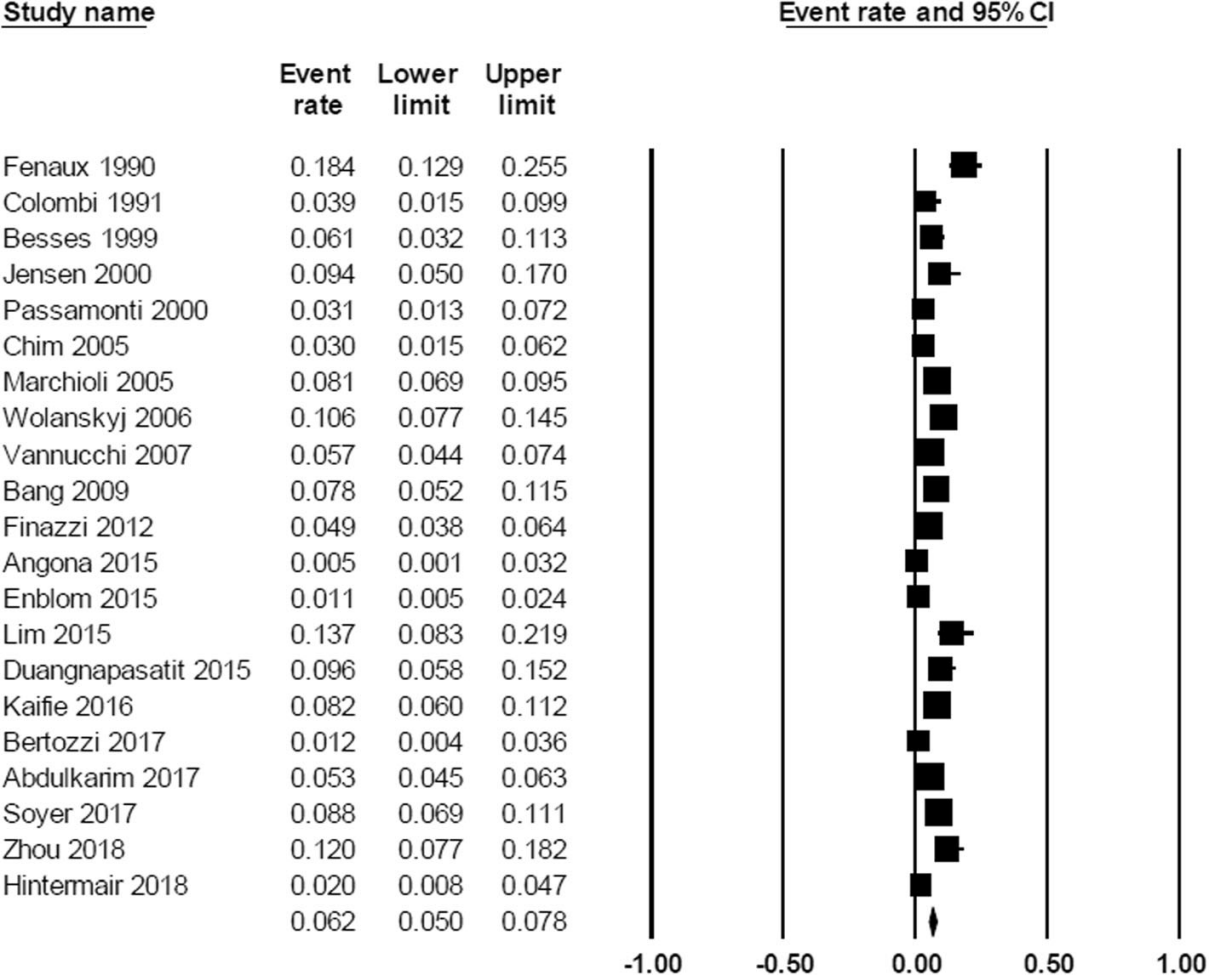
Forest plot of pooled prevalence and 95% confidence interval of overall bleeding complications in patients with MPN

**Fig. 6:**
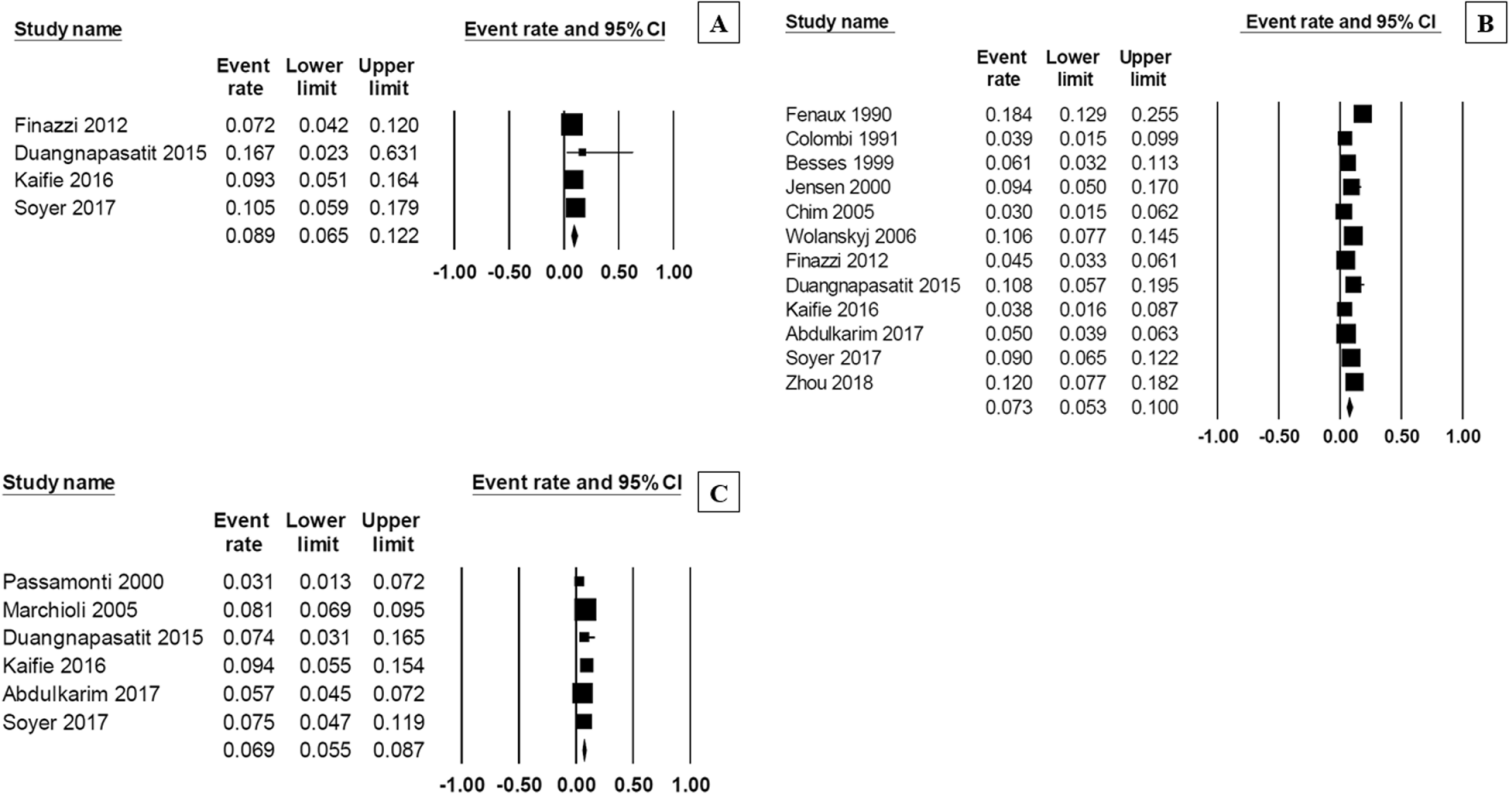
Forest plot of pooled prevalence and 95% confidence interval of overall bleeding complications of each MPN subtype: **a** primary myelofibrosis; (**b**) essential thrombocythemia; (**c**) polycythemia vera

The pooled prevalence of hemorrhagic complications among patients who were newly diagnosed with MPN patients was 6.2% (95% CI, 5.0–7.8%; I^2^ 85%; Fig. 5) [7–9, 11–14, 16, 18, 19, 24–29, 31–35]. The pooled prevalence for each MPN subtype was as followed; PMF 8.9% (95% CI, 6.5–12.2%; I^2^ 0%) [24, 28, 29, 32], ET 7.3% (95% CI, 5.3–10.0%; I^2^ 84%) [7–9, 11, 13, 16, 24, 28, 29, 31, 32, 34], and PV 6.9% (95% CI, 5.5–8.7%; I^2^ 53%) (Fig. 6) [12, 14, 28, 29, 31, 32].

Prevalence of some specific types of bleeding at the diagnosis of MPN was reported by the included studies. Their pooled prevalence was as followed; mucocutaneous bleeding 2.8% (95% CI, 1.4–5.8%; I^2^ 35%) [11, 19, 28], gastrointestinal bleeding 2.1% (95% CI, 1.3–3.1%; I^2^ 68%) [7, 8, 11, 13, 24, 26, 28, 29, 31, 35], epistaxis 1.0% (95% CI, 0.5–2.0%; I^2^ 0%) [8, 11, 26], and postoperative bleeding 1.1% (95% CI, 0.5–2.1%; I^2^ 0%) [8, 11, 28, 29]. The forest plots of each specific type of bleeding are provided as Additional file 5.

## Discussion

The current study is the first systematic review and meta-analysis to evaluate the frequency of thrombotic and hemorrhagic events as the initial manifestations of MPN. We found the pooled prevalence of thrombosis at diagnosis of MPN of 20% although the prevalence from individual study varied considerably, ranging from 9.5 to 38.6%. Thrombosis could be on either arterial or venous site and appeared to be more common among patients who were newly diagnosed with PV than ET and PMF. The pathogenesis of acquired thrombophilic state in these patients is probably multifactorial in nature. Two main mechanisms have been proposed. One involves abnormalities of platelets, leukocytes, and red blood cells arising from the clonal hematopoietic cell proliferation. These abnormal cells interact and activate coagulation pathway more often than normal cells, leading to chronic activation of the coagulation cascade. Another postulated mechanism involves the chronic inflammatory state of MPN as studies have demonstrated that inflammatory cytokines can cross-activate coagulation factors and inhibit fibrinolytic pathway. In addition, those cytokines and reactive oxygen species are known to post deleterious effect on vascular endothelial cells, resulting in vascular injury. Both would serve as the fundamental factors for increased clotting tendency [39–41].

On the other hand, a high prevalence of hemorrhage at diagnosis was also observed in patients with MPN, although lower than the prevalence of thrombosis. The pathogenesis of hemorrhagic complications among patients with MPN is also probably multifactorial but it is believed that acquired von Willebrand disease from excessive binding of von Willebrand factor with the abnormal platelets and increased von Willebrand factor proteolysis is the most likely major player [42, 43]. In fact, the pattern of bleeding among patients with MPN, including gastrointestinal, mucosal, and cutaneous bleeding, is quite similar to von Willebrand disease [44]. Other possible contributing factors include thrombocytopenia from bone marrow failure associated with advance disease and secondary hemostatic defects from liver impairment due to liver fibrosis and extramedullary hematopoiesis associated with PMF [42].

The high frequency of both thrombotic and hemorrhagic events among patients with MPN demonstrated by this study has some clinical implications. First, both thrombosis and bleeding are common initial manifestations of MPN. Therefore, investigations for MPN may be warranted for patients who present with unexplained thrombosis or abnormal bleeding. Second, patients with MPN should be considered as those at higher risk of thrombotic complication and, therefore, prophylaxis with anti-platelet and/or anti-coagulation may provide benefit. Nonetheless, these patients are also at a higher risk of hemorrhagic complication and the potential benefit of thrombotic prophylaxis needs to be balanced with the bleeding risk especially in patient with extreme thrombocytosis. Further studies are still needed before the final recommendations can be made.

There are some limitations in this study. First, it is a meta-analysis of descriptive studies that reported the prevalence of thrombosis and bleeding among patients with MPN. There were no subjects without MPN to serve as controls and, therefore, this study could not provide the information on the magnitude of the risk relative to general population. Second, between-study heterogeneity was high in most analyses which was probably due to the fact that the included studies were conducted over the time span of the past three decades and the diagnostic criteria for each MPN subtype have evolved during that time. To illustrate, based on the 2001 World Health Organization (WHO) classification of MPNs, hemoglobin level > 18.5 g/dL in man and > 16.5 g/dL in woman were the criteria for the diagnosis of PV and one of criteria for the diagnosis of ET was platelet count ≥600 × 10^9^/L [45]. However, the cut-offs for diagnosis of PV and ET in the 2016 WHO classification of MPNs were lowered to the hemoglobin levels of > 16.5 g/dL in man and > 16 g/dL in woman and platelet count of ≥450 × 10^9^/L, respectively [2]. Therefore, some cases of PV and ET could have been missed by the previous classification criteria. As a result, it is likely that the characteristics of the diseases are different between the older and newer studies, even though they are labeled with the same name. It is also likely that the differences in the technology used to identify thrombosis and bleeding among these patients during the period would have affected the prevalence.

## Conclusions

Thrombosis and bleeding are common initial manifestations of MPN. Investigations for MPN should be considered for patients who present with unexplained thrombosis or abnormal bleeding.

## Additional files

Additional file 1: Search strategy. (DOCX 15 kb)

Additional file 2: The Preferred Reporting Items for Systematic Reviews and Meta-Analyses statement. (DOC 63 kb)

Additional file 3: **Table S1.** Managements and clinical outcomes of each included study. (DOCX 20 kb)

Additional file 4: Forest plots of pooled prevalence and 95% confidence interval of each type of thrombosis in the patients with MPN. (DOCX 1022 kb)

Additional file 5: Forest plot of pooled prevalence and 95% confidence interval of each type of bleeding in the patients with MPN. (DOCX 441 kb)

## Abbreviations

ET: Essential thrombocythemia
MPNs: Myeloproliferative neoplasms
PE: Pulmonary embolism
PMF: Primary myelofibrosis
PV: Polycythemia vera
WHO: World Health Organization
