# Supplementary material for: A systematic review and meta-analysis of the prevalence of thrombosis and bleeding at diagnosis of Philadelphia-negative myeloproliferative neoplasms
Source: BMC Cancer. 2019 Feb 28;19:184. doi: 10.1186/s12885-019-5387-9 (PMC6393965; doi:10.1186/s12885-019-5387-9)
Supplement: Supplementary file 1 — Search strategy. (DOCX 15 kb) [file 12885_2019_5387_MOESM1_ESM.docx]

**Additional file 1**

Search strategy

**EMBASE**

1. 'myeloproliferative'/exp OR 'myeloproliferative'
2. 'thrombosis'/exp OR 'thrombosis'
3. 'bleeding'/exp OR 'bleeding'
4. #2 OR #3
5. #1 AND #4

**Medline**

1. myeloproliferative.mp. or exp Myeloproliferative/
2. thrombosis.mp. or exp Thrombosis/
3. bleeding.mp. or exp Bleeding/
4. 2 or 3
5. 1 and 4
