# Supplementary material for: A systematic review and meta-analysis of the prevalence of thrombosis and bleeding at diagnosis of Philadelphia-negative myeloproliferative neoplasms
Source: BMC Cancer. 2019 Feb 28;19:184. doi: 10.1186/s12885-019-5387-9 (PMC6393965; doi:10.1186/s12885-019-5387-9)
Supplement: Supplementary file 3 — Table S1. Managements and clinical outcomes of each included study. (DOCX 20 kb) [file 12885_2019_5387_MOESM3_ESM.docx]

**Table S1** Managements and clinical outcomes of each included study

| **References** | **MPN treatments** | **Clinical outcomes during follow-up** |
| --- | --- | --- |
| **Fenaux 1990 [7]** | Antiplatelets (40.8%), HU (48.9%), Bu (23.8%), ^32^P (14.9%) | 5-year OS (all cases) 81%  Thrombotic events: 22 patients |
| **Colombi 1991[8]** | Antiplatelets, chemotherapy with HU, Mel, Bu | 4-year OS (all cases) 89.3% |
| **Besses 1999 [9]** | Dipyridamole (32.8%), ASA (50%), HU (54.0%), ^32^P (40.2%), Bu (4.9%), Mel (0.8%) | 5-year OS (all cases) 89.9%  Thrombotic events: 24 patients  Bleeding events: 17 patients |
| **Manoharan 1999 [10]** | NR | Thrombotic events: 11 evens |
| **Jensen 2000 [11]** | ASA (70%), anagrelide (2%), anticoagulants (3%), HU (95%), Bu/ Mel/ Chlorambucil (20%) | 5-year OS (all cases) 69%  Thrombohemorrhagic events: 55 episodes in 37 patients |
| **Passamonti 2000 [12]** | Antiplatelets (34%), phlebotomy (16%), pipobroman (100%), Bu (1.8%), HU (1.8%) | 10-year OS (all cases) 78%  Thrombosis rate during follow-up 14% |
| **Chim 2005 [13]** | Anagrelide (1.5%), HU (93.3%), ^32^P (2.2%), Mel (2.9%) | 5-year OS (all cases) 80%  10-year TFS 60%  10-year BFS 83%  Thrombotic events: 23 events  Bleeding events: 15 events |
| **Marchioli 2005 [14]** | Antiplatelets (58.3%), anticoagulants (6.7%), phlebotomy (63.5%), HU (48.4%), IFN-α (3.9%), Bu (3.7%), pipobroman (6.5%), chlorambucil (0.3%), ^32^P (2.7%) | 5-year OS (all cases) 90%  Cumulative rate of thrombotic events: 5.5 events/100 persons/year  Cumulative rate of total bleeding events: 2.9 events/100 persons/year |
| **References** | **MPN treatments** | **Clinical outcomes during follow-up** |
| **Cervantes 2006 [15]** | HU (40.3%), androgen (33%), danazol (30.2%), oxymetholone (2.8%), ESA (18.3%), prednisolone (13.8%), 6-MP (9.2%), anagrelide (4.6%), IFN-α (4.6%) | Median survival time 4.1 years  4-year thrombotic event 11.6%  5-year TFS 90.4% |
| **Wolanskyj 2006 [16]** | ASA (62.4%), cytoreductive agents (82.9%) | Median survival time 18.9 years |
| **Carobbio 2007 [17]** | Antiplatelets (47%), HU (52%) | Thrombotic events: 78 episodes in 67 patients |
| **Vannucchi 2007 [18]** | Phlebotomy (22.3%), chemotherapy (51.8%) | Thrombotic events: 122 patients |
| **Bang 2009 [19]** | NR | 4-year OS (all cases) 95.8%  Thrombotic events: 87 episodes in 80 patients  Bleeding events: 39 episodes in 37 patients  No thrombotic and bleeding related-mortality |
| **Elliott 2010 [21]** | Antiplatelets, anticoagulants | Thrombotic events: 22 patients |
| **Palandri 2011 [22]** | ASA (92%), cytoreductive agents (86%) | 2-year OS 77% |
| **Buxhofer-Ausch 2012 [23]** | ASA (73.6%), cytoreductive agents (71.6%) | 6-year thrombotic rate 20.8% |
| **Finazzi 2012 [24]** | ASA, cytoreductive agents | NR |
| **Angona 2015 [25]** | NR | 5-year OS 94.8%  5-year TFS 87.5% |
| **Enblom 2015 [26]** | NR | NR |
| **Lim 2015 [27]** | Phlebotomy (34.3%), antiplatelets (91.2%), HU (80.4%), anagrelide (18.6%) | NR |
| **Duangnapasatit 2015 [28]** | NR | Thrombotic events: 5 patients |
| **Kaifie 2016 [29]** | ASA (57.4%), anagrelide (15.1%), warfarin (10%), rivaroxaban (1.8%), HU (49.2%), ruxolitinib (19.9%), iMID (4.8%), IFN-α (9.2%), SCT (4.6%) | Bleeding events: 34 events |
| **Cerquozzi 2017 [30]** | Phlebotomy (80%), ASA (74%), cytoreductive agents (82%) | Thrombotic event rate 2.4/ 100 persons/year |
| **References** | **MPN treatments** | **Clinical outcomes during follow-up** |
| **Abdulkarim 2017 [31]** | **PV**: Low-dose ASA (59%), warfarin or clopidrogel (4%), HU (42.4%), IFN-α (3.9%), Bu (0.5%), ^32^P (1.4%)  **ET**: Low-dose ASA (59%), other anticoagulants (2%), HU (60.9%), IFN-α (4.3%), Bu (0.5%), ^32^P (1.8%) | NR |
| **Soyer 2017 [32]** | Antiplatelets (78.1%), HU (89.6%), anagrelide (4.8%), IFN-α (5.6%) | 10-year OS (all cases) 86.7% |
| **Bertozzi 2017 [33]** | Low-dose ASA, LMWH, warfarin | Thrombotic events: 55 patients  Bleeding events: 27 patients |
| **Zhou 2018 [34]** | NR | 2-year thrombotic rate 16.8% |
| **Hintermair 2018 [35]** | **PV**: ASA (84.8%), phlebotomy (72.7%), HU (75.8%), ruxolitinib (3%), IFN-α (15.2%), anagrelide (18.2%)  **ET**: ASA (73.3%), HU (66.7%), IFN-α (13.3%), anagrelide (46.7%)  **PMF**: ASA (33.3%) | Thrombotic events:11 events |

**Abbreviations:** ASA: Aspirin; BFS: Bleeding-free survival; Bu: Busulfan; ESA: Erythroid stimulating agent; ET: Essential thrombocythemia; HU: Hydroxyurea; IFN-α: Interferon-alpha; LMWH: Low molecular weight heparin; NR: Not reported; Mel: Melphalan; Not reported; OS: Overall survival; PMF: Primary myelofibrosis; PV: Polycythemia vera; SCT: Stem cell transplant: TFS: Thrombotic-free survival; 6-MP: 6-mercaptopurine; ^32^P: Radiophosphorous
