# Supplementary material for: A systematic review and meta-analysis of the prevalence of thrombosis and bleeding at diagnosis of Philadelphia-negative myeloproliferative neoplasms
Source: BMC Cancer. 2019 Feb 28;19:184. doi: 10.1186/s12885-019-5387-9 (PMC6393965; doi:10.1186/s12885-019-5387-9)
Supplement: Supplementary file 4 — Forest plots of pooled prevalence and 95% confidence interval of each type of thrombosis in the patients with MPN. (DOCX 1022 kb) [file 12885_2019_5387_MOESM4_ESM.docx]

**
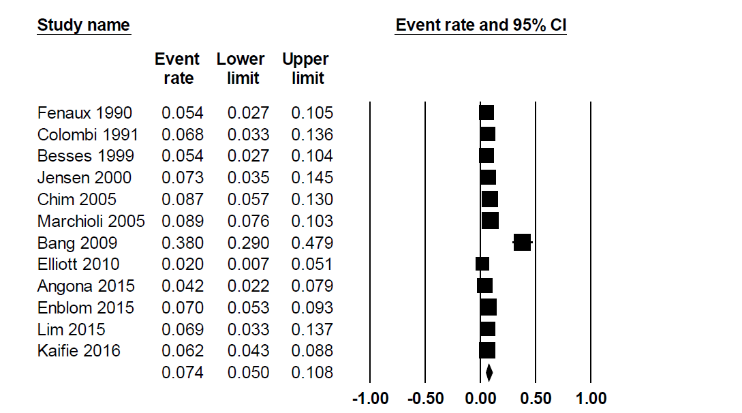

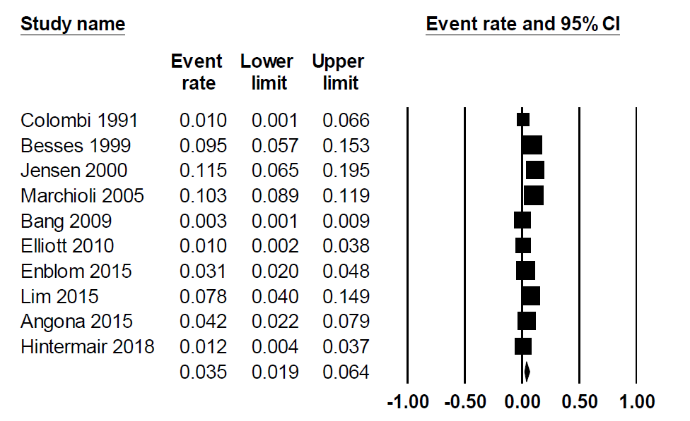
**

**D**

**B**

**C**

**A**

**
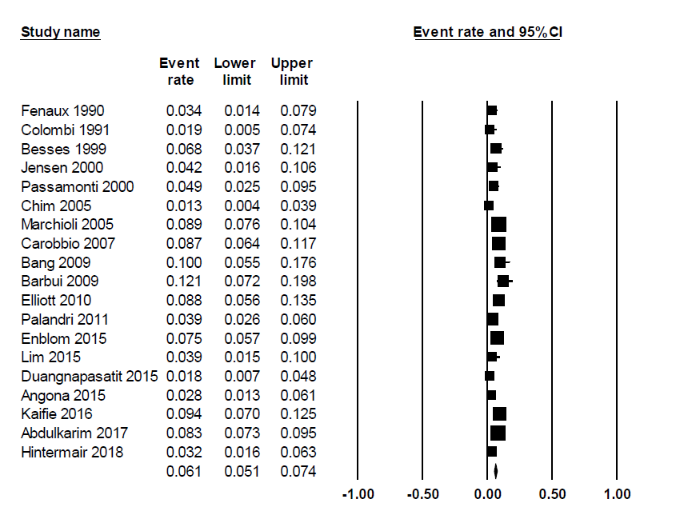

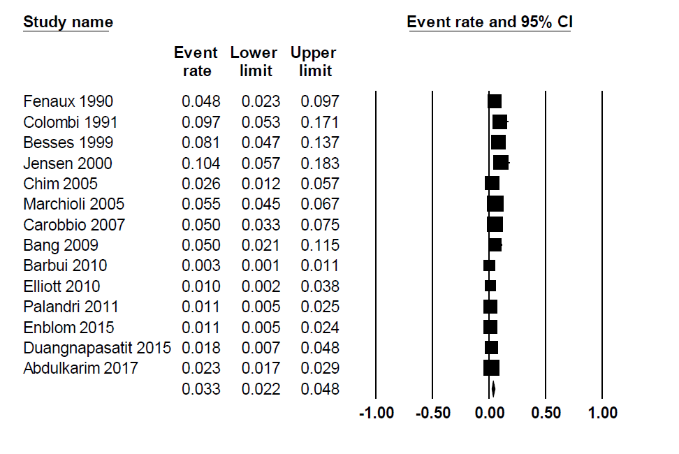
**

**F**

**
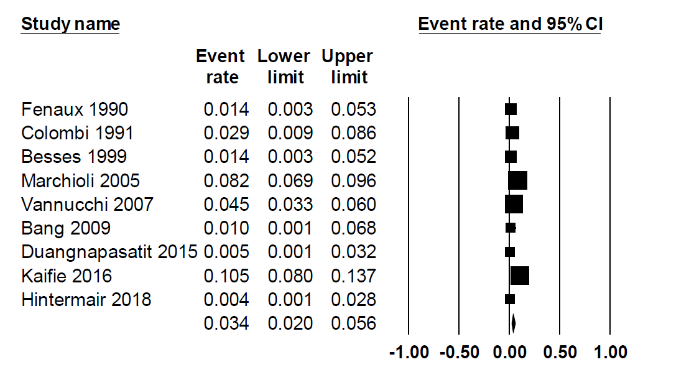

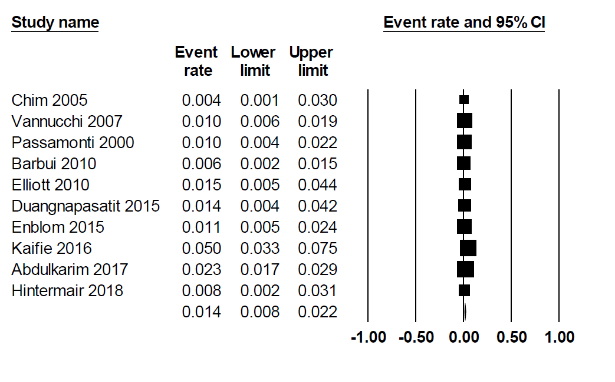
**

**H**

**G**

**E**

**
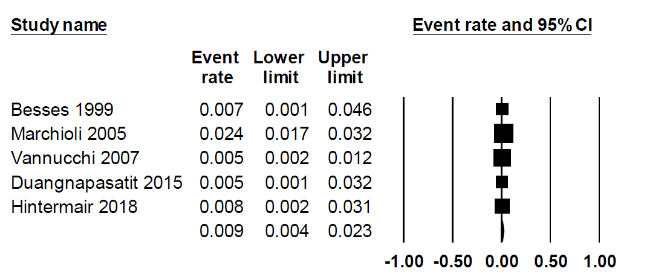

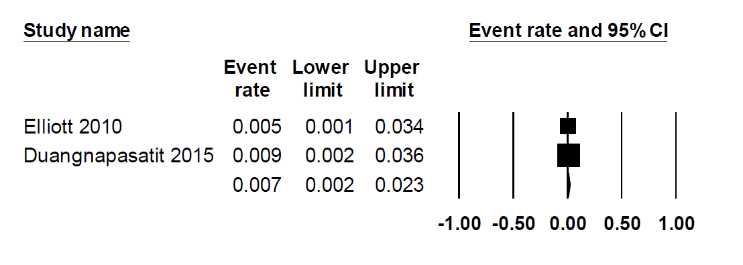
**

**Additional file 4** Forest plots of pooled prevalence and 95% confidence interval of each type of thrombosis in the patients with MPN: (**a**) ischemic stroke; (**b**) transient ischemic attack; (**c**) coronary heart disease; (**d)** peripheral arterial disease; (**e**) deep vein thrombosis; (**f**) splanchnic vein thrombosis; (**g**) pulmonary embolism; (**h**) cerebral venous sinus thrombosis
