# Supplementary material for: A systematic review and meta-analysis of the prevalence of thrombosis and bleeding at diagnosis of Philadelphia-negative myeloproliferative neoplasms
Source: BMC Cancer. 2019 Feb 28;19:184. doi: 10.1186/s12885-019-5387-9 (PMC6393965; doi:10.1186/s12885-019-5387-9)
Supplement: Supplementary file 5 — Forest plot of pooled prevalence and 95% confidence interval of each type of bleeding in the patients with MPN. (DOCX 441 kb) [file 12885_2019_5387_MOESM5_ESM.docx]

**
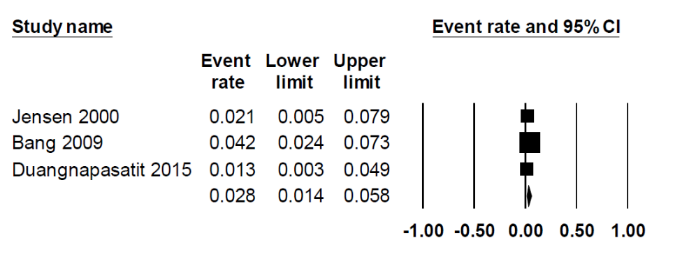

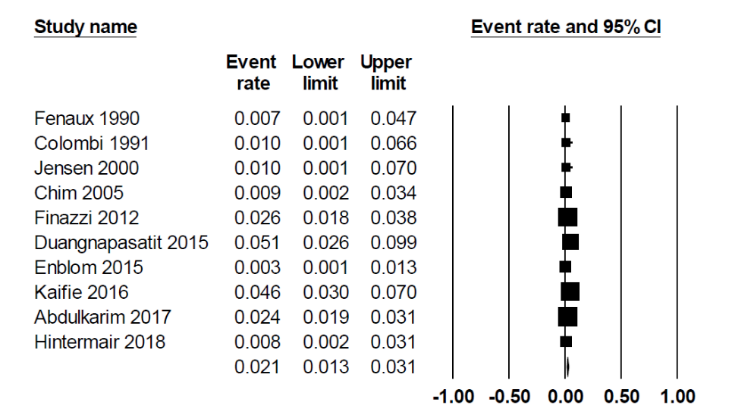
**

**A**

**B**

**
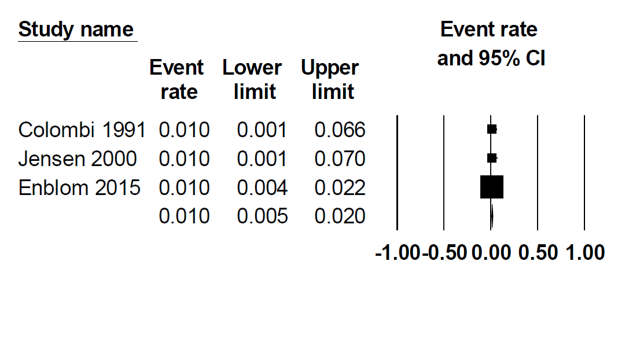

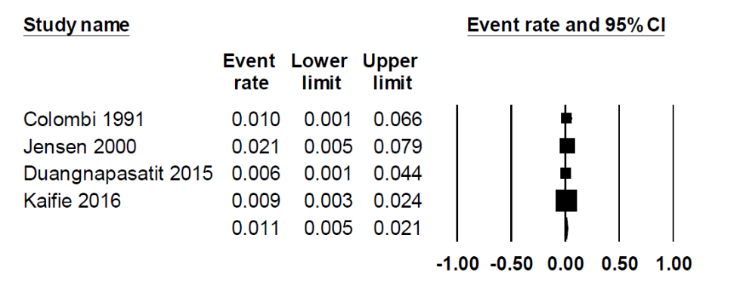
**

**D**

**C**

**Additional file 5** Forest plot of pooled prevalence and 95% confidence interval of each type of bleeding in the patients with MPN: (**a**) mucocutaneous bleeding; (**b**) gastrointestinal bleeding; (**c**) epistaxis; (**d**) postoperative bleeding
